# Lumbar Intervertebral Disc and Discovertebral Segment, Part 1: An Imaging Review of Normal Anatomy

**DOI:** 10.7759/cureus.25558

**Published:** 2022-06-01

**Authors:** Daphne J Theodorou, Stavroula J Theodorou, Ioannis D Gelalis, Yousuke Kakitsubata

**Affiliations:** 1 Radiology, MRI-CT Unit, General Hospital of Ioannina, Greece, Ioannina, GRC; 2 Radiology, Musculoskeletal MRI, University Hospital of Ioannina, Ioannina, GRC; 3 Orthopaedics, University Hospital of Ioannina, Ioannina, GRC; 4 Radiology, Musculoskeletal Imaging, Miyazaki Konan Hospital, Miyazaki, JPN

**Keywords:** cadavers, lumbar spine, correlative study, multimodality imaging, normal anatomy, discovertebral segment, disc anatomy, intervertebral disc

## Abstract

The intervertebral disc is designated the most important cartilaginous articulation of the vertebral column that functions to withstand compressive biomechanical forces and confer strength and flexibility to the spine. A thorough study of the complex fine structure and anatomic relationships of the intervertebral disc is essential for the characterization of the integrity of each individual structure in the discovertebral segment. This elaborate work in human cadavers explores the sophisticated internal structure of the normal intervertebral disc and the discovertebral segment, providing detailed data derived from the dissection of specimens through imaging and close anatomic-histologic correlation. Familiarity with the normal appearances and basic functional properties of the lumbar intervertebral disc and discovertebral segment is fundamental for the recognition of aberrations that may have important clinical implications in patients with low back pain. In Part I of this article, the anatomic structure and features of the discovertebral complex in adults will be described.

## Introduction

The intervertebral disc is a fibrocartilaginous structure that forms a symphysis with the adjacent vertebra, comprising the discovertebral segment. Although intuitively deemed a simple, bi-component structure that is comprised of the centrally located nucleus pulposus surrounded by the annulus fibrosus, in virtue it is a highly sophisticated structure with intricate hydraulic biomechanical properties [1] that enable maintenance of structural integrity in the spine under physiological load [2]. Degenerative changes affecting the intervertebral disc per se and the discovertebral segment have been documented in the imaging literature as a significant source of pain, especially in middle-aged and elderly persons [3]. Indeed, a plethora of distinct pathologic processes can impair the fine structural anatomy of the intervertebral disc and discovertebral segment, ranging from osseous and cartilaginous changes to those in surrounding soft tissue [4-6]. Knowledge of normal imaging anatomy of the intervertebral disc and discovertebral segment is considered sine qua non in recognition of the many pathologic conditions that can become a potential major source of symptoms in the spine. 

Studies in human cadavers provide solid evidence of a highly sophisticated internal structure of the intervertebral disc [7]. This imaging review article delineates the normal anatomy of this seemingly simple-yet fine anatomic structure, the lumbar disc, using multimodality imaging. In so doing, documentation of the imaging findings is feasible through close correlation with findings derived from an anatomic inspection of the dissected cadaveric specimens. An emphasis has been placed on the MR imaging findings we have encountered through close cross-sectional anatomic and histologic characterization, building the foundation of medical diagnosis. This presentation is helpful, as detailed knowledge of the morphological intrinsic characteristics of the intervertebral disc and discovertebral segment can have implications for advanced treatment related to developing regenerative therapies through the cell and tissue-engineering approaches [1].

## Materials and methods

Cadavers: specimens and preparation

Lumbar spine columns were harvested from 65 fresh-frozen cadavers in one piece with a saw. Ages at death ranged from 29 weeks of gestation to 90 years (mean 70 years). All specimens contained the vertebral bodies from L1 through S1, resulting in a total of 325 intervertebral spaces, of which 320 were intact. Excessive paraspinal soft tissues were removed. All cadaveric spines were examined with conventional radiography to determine the presence or absence of surgical intervention or instrumentation, degenerative changes, rheumatologic disorders, vertebral fractures, malignancy, and gross destruction of the vertebral end plate. The specimens were deep-frozen at −80°C for 48 hours and then allowed to thaw for 24 hours at room temperature prior to the multimodal imaging studies.

MR imaging assessment 

MR images were obtained with 1.5-T superconducting MR units (MRT 200/RX, Toshiba, Tokyo, Japan; Signa, GE Medical Systems, Milwaukee, Wisconsin). A standard receive-only head or knee coil was used for imaging the entire lumbar spine in the supine position, and three-inch surface dual coils were used for imaging individual intervertebral discs. MR images were acquired in the sagittal and axial planes. For MR discography, images with fat suppression were acquired with the same technical parameters as those used in pre-contrast MR imaging studies. CT images were obtained in the axial- and were reformatted in the sagittal plane. Gray scale sonography was performed at the level of each intervertebral disc in axial and sagittal planes with 10-MHz transducers (PowerVision; Toshiba Medical Systems Co., Ltd., Tokyo, Japan).

After imaging, cadaveric specimens were deep-frozen again for 72 hours and subsequently cut into 3 mm-thick sagittal slabs with a band saw so that anatomic sections corresponded closely to MR scans. Low-kilovoltage contact radiographs (55 KVp, 0.3 mA) of each section were obtained with an x-ray unit with fine grain film processed in a normal manner. The images derived from each of the methods (radiography, computed tomography, MR imaging, MR discography, Faxitron radiography, and sonography) were evaluated in a random order and in close correlation to the findings on macroscopic inspection of the anatomic slices. Finally, the slices were decalcified in 20% formic acid, and histologic sections were prepared with hematoxylin-eosin (HE), Elastica van Gieson, toluidine blue (pH=7), and Mallory-Azan staining. For each of the discovertebral segments, data derived from multimodality imaging were closely correlated to those of the anatomic sections.

## Results

The intervertebral disc: biochemical considerations

Differences in the biochemical and histologic composition of the two distinct disc components grant unique biomechanical properties to each of these parts. The annulus fibrosus contains a rich network of collagen fibrils arranged in a lamellar pattern. Regional differences in the structure of the annulus account for the stiffness of its peripheral portion enduring resistance to axial and torsional stress [8]. The nucleus pulposus, a gelatinous structure containing loose collagen fibers suspended in a mucoprotein matrix, is located eccentrically closer to the posterior semi-circumference of the disc. Both the annulus fibrosus and the nucleus pulposus are composed of water and proteoglycans (PGs), in different proportions. Proteoglycan molecules (similar to those in the articular cartilage) can attract, absorb, and retain water in the disc and are found in greater amounts within the nucleus pulposus. The PGs generate osmotic pressure that keeps the disc expanded through a shift of extracellular fluid from the outside to the inside of the nucleus pulposus [9,10]. This highly sophisticated internal structure and the macroscopic appearance of the disc change with age. However, in young individuals, the resilient nucleus pulposus has a gel-like consistency and is easily discernible from the annulus fibrosus. Because of a decrease in the amount of water and PGs and the chondroitin-keratan sulfate ratios with advancing age, the aging nucleus pulposus is gradually replaced by fibrocartilage, assuming a stiffer consistency, and it is virtually difficult to differentiate it from the surrounding annulus fibrosus [9,11].

With aging, a cascade of biochemical alterations including dehydration of the matrix, changes in proteoglycan composition with an accumulation of metalloproteinases from the degrading collagen, and cellular senescence lead to appreciable morphologic changes in the discovertebral segment associated with alterations in the mechanical properties of tissue [3,10,12]. Whether aging-related changes and degeneration are separate processes or the same process occurring over a different timescale is currently a matter of great debate and speculation [13,14]. 

The intervertebral disc and discovertebral segment: anatomic considerations

**Figure 1:**
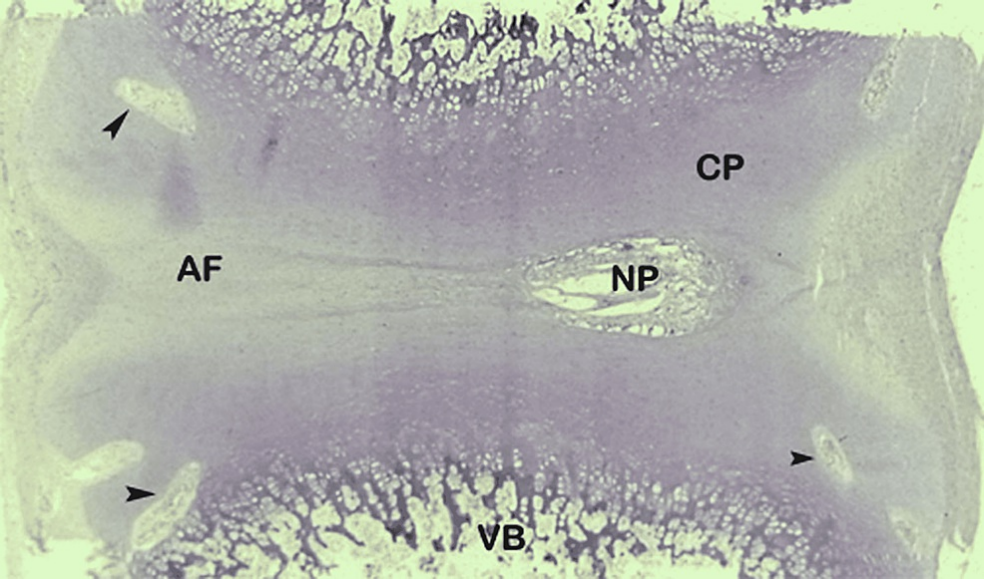
Sagittal anatomic section of 29-week-old premature fetus lumbar disc. Boundary between nucleus pulposus (NP) and annulus fibrosus (AF) is distinct. There are notochordal remnants in the nucleus, which contain abundant amorphous mucoid material. The notochordal area is surrounded by fibrocartilage and the annulus fibrosus has a collagenous consistency. Cartilaginous end plates (CPs) cover entirely superior and inferior surfaces of the vertebral body (VB). Vascular channels are seen in hyaline cartilage (arrowheads).

**Figure 2:**
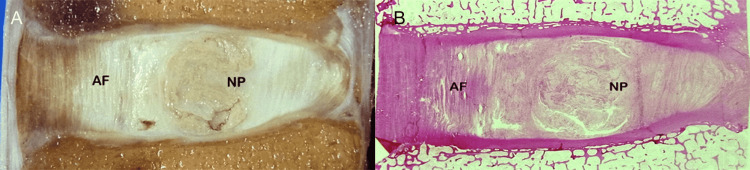
Normal disc. Morphology. (A) Photograph of the sagittal section of the spine shows the nucleus pulposus (NP) surrounded by annulus fibrosus (AF). (B) Corresponding histology slice delineates the anatomy of the normal disc.

Formation of the embryonic intervertebral disc is a highly-coordinated developmental process that is ruled by numerous genes and regulatory molecules. At the embryonic level, the annulus fibrosus and the cartilaginous end plate are derived from the sclerotome, whereas the nucleus pulposus originates from the endoderm, a remnant of the notochord [11]. In the premature fetal spine, there are abundant notochordal cells hosted in the disc. Although in developing discs, the nucleus pulposus and annulus fibrosus are sharply demarcated (Figure 1), the prevalence of these findings decreases in older persons [15,16]. With advancing age, cells producing collagen are sparse and chondrocyte-like cells increase [11]. The demarcation between the nucleus and annulus is less distinct from the second decade onwards as the nucleus pulposus becomes increasingly more fibrous [11,12]. According to our focused evaluation, the lumbar intervertebral disc itself is a discrete, bi-component anatomic structure contained in the intervertebral space (Figure 2).

**Figure 3:**
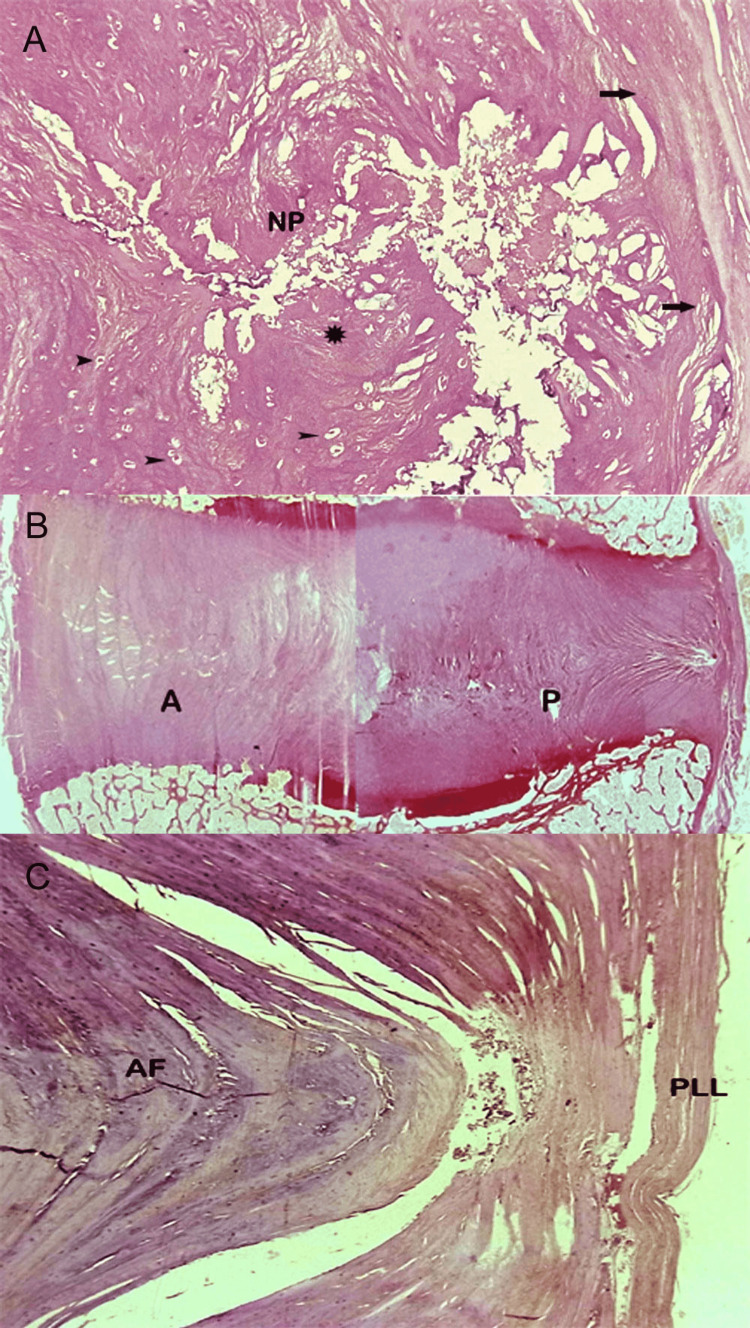
Normal disc. Histologic features. (A) Histologic section of the gelatinous nucleus pulposus (NP) comprised of fibrous tissue (asterisk) and cells (arrowheads). Note peripheral fiber bundles of nucleus blending with fibrocartilage of annulus (arrow) (H&E, objective Χ20). (B) Histologic section of the annulus fibrosus (composite image) delineates the intricate internal structure and arrangement of lamellae in the anterior (A) and posterior (P) portions of the annulus fibrosus. (C) Histologic section of the posterior portion of annulus fibrosus (AF). Note thickening of most posterior annular fibers at the site of attachment of the dense posterior longitudinal ligament (PLL) (H&E, objective Χ20).

The nucleus pulposus is a semi-liquid structure that occupies an eccentric position with regard to the vertical midline axis of the vertebral body, closer to the posterior border of the intervertebral disc. The annulus fibrosus is a multi-lamellar structure (contains 15 to 25 lamellae) engulfing the nucleus pulposus in an elliptical, reniform arrangement [4,12]. The lamellae at the posterior portion of the disc are fewer in number as compared to those in the anterior portion, a structural feature that appears to bear implications on the pathogenesis of posterior disc displacement [3]. Our anatomic analysis revealed that the posterior lamellae of the annulus fibrosus are also noticeably thinner and more densely packed as compared to the lamellae in the anterior-most portion of the disc. On the basis of analysis of histologic findings, the peripheral zone of the annulus at the outermost border of the disc is comprised of distinct collagenous fibers [16]. More inwards, fibers blend imperceptibly and farther centrally, close to the nucleus pulposus, the inner zone is comprised of fibrocartilage. The former, outermost portion of the annulus fibrosus is thicker and stronger posteriorly, in the region where it receives a broad reinforcing attachment from the vertical posterior longitudinal ligament (Figure 3).

Less thickening of the outer fibers in the anterolateral surface of the annulus fibrosus is seen at the site of wide insertion of the anterior longitudinal ligament. We have noted overt pathologic changes of degeneration, calcification, laxity, and rupture of the longitudinal ligaments on anatomic inspection of the specimens in our study. Such abnormal morphologic alterations, resulting in structurally weaker ligaments, may play an indisputable role in the pathogenesis of disc displacement.

**Figure 4:**
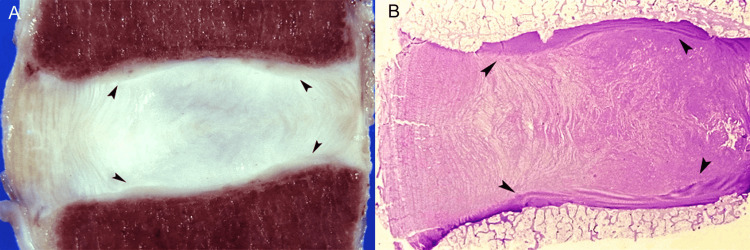
Anatomy of cartilaginous end plates. (A) Anatomic section shows cartilaginous end plates (arrowheads). (B) Corresponding histology section depicts cartilaginous end plates (arrowheads) of variable thickness.

At the discovertebral junction, the outermost anterior fibers of the annulus fibrosus are fixed to the anterior surface of the vertebral body by firm Sharpey fibers [16,17]. In the vertebral end plates, the hyaline cartilaginous end plates lay on the true epiphyseal plates, the latter be bone plates. End plate morphology ranges from a thick and well-defined layer of bone lined by hyaline cartilage that is calcified in its basal surface where it comes in direct contact with the underlying bone to a thin, eroded, discontinuous and perforated subchondral bone plate that allows direct contact of bone marrow and nonmineralized cartilage (Figure 4).

An increased frequency of degenerative lesions with advancing age was documented, in our specimens. The concept of progression of degenerative change to breach in the cortical bone underscores the difficulty encountered when trying to distinguish among degenerative, traumatic, or malignant invasion of the end plates and, moreover, indicates that a spectrum of pathologic conditions may exist in many cases. 

**Figure 5:**
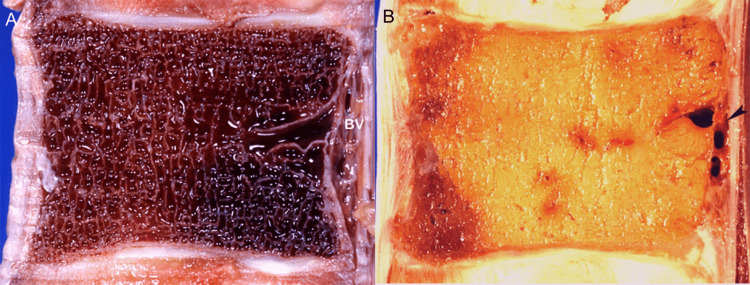
Vertebral bone marrow age-related changes. (A) Anatomic section of cellular (red) marrow. Note basivertebral vein (BV). (B) Anatomic section of fat (yellow) marrow.

The vertebral bone marrow varies in cellular composition and appearance according to age. At birth, metabolically active red marrow (composed of 60% hematopoietic cells and 40% fat cells) occupies the entire vertebral body, and with aging, it is progressively replaced by metabolically inactive yellow marrow (composed of 95% fat cells and 5% nonfat cells) (Figure 5).

**Figure 6:**
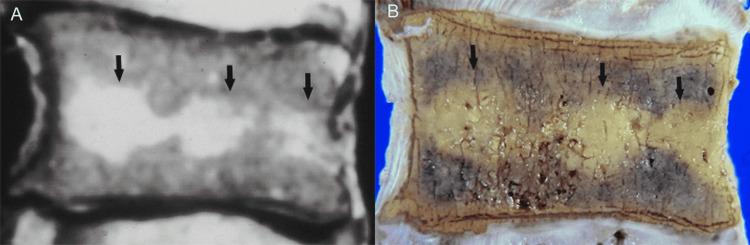
Vertebral marrow conversion. (A) Sagittal T2-weighted MR image shows a high signal intensity zone traversing the center of the vertebral body (arrows). (B) Corresponding anatomic section shows fatty marrow (arrows) in the above-mentioned distribution.

On MR images, the red marrow exhibits intermediate signal intensity on both T1- and T2-weighted images, while the yellow marrow shows a high signal on T1- and decreased signal intensity on conventional T2-weighted images. During the conversion process, the pattern of marrow distribution changes, and yellow marrow forms islands inside red marrow, typically situated around the basivertebral vein. We have found a similar presentation where heterogenous red marrow was predominantly located close to the vertebral end plates and yellow marrow was identified around basilar veins (Figure 6).

The blood supply of the intervertebral disc remains subject to debate [18,19]. At birth, the disc has some vascular supply, but these vessels soon recede, leaving the disc with little direct blood supply in the physiologic adult [12]. The central component, the nucleus, is avascular. Accordingly, the metabolism of the disc changes largely with age, and the nutrition of the disc becomes dependent on the diffusion of fluid, either from the marrow through the subchondral bone or the cartilaginous end plate, or from neighboring blood vessels through the annulus fibrosus [3,18-22].

Although the annulus fibrosus until recently also has been considered to be avascular, new evidence suggests otherwise. It appears that observations of vascular ingrowth into the inner layers of the annulus fibrosus and the cartilaginous end plates associated with aging and degeneration may have important implications with regard to tissue healing after surgical reconstructive procedures [10,11-14,20-22]. Such vascularity was not documented in normal discs in our investigation, however. We only found vascular ingrowth in the discs and cartilaginous end plates of specimens associated with gross pathology in the presence of annular tear, fracture, or malignancy [23]. 

Pathologic and imaging correlation: the concept of exact structural designation

**Figure 7:**
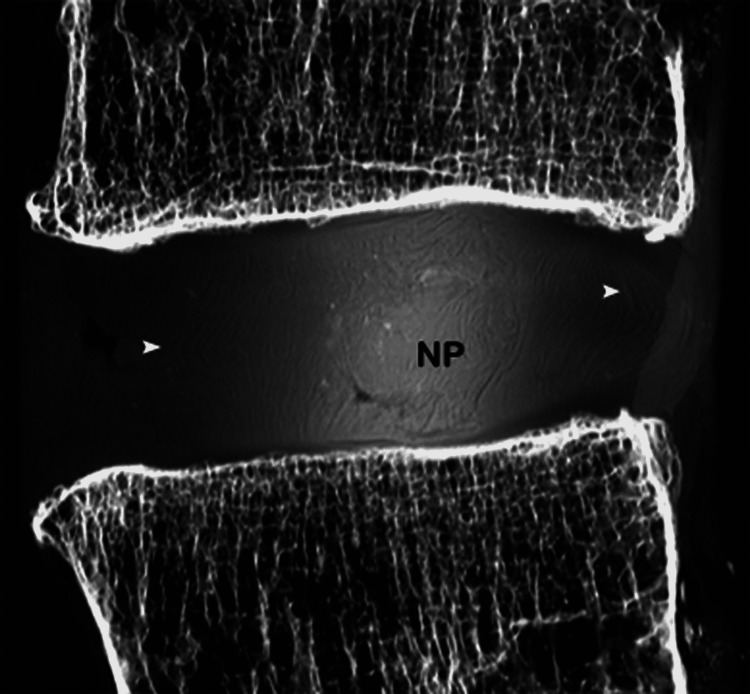
Radiography. High-quality (Faxitron) radiograph in a sagittal anatomic slice of a cadaveric specimen depicts the internal structure of the disc. The nucleus is clearly outlined and the concentric arrangement of annular fibers is visible (arrowhead). NP: nucleus pulposus.

High-quality, cross-sectional imaging has revolutionized the evaluation of the spine, allowing for noninvasive visualization of the osseous and soft tissue structures [23]. Although the characterization and function of the anatomic structures have remained constant in the literature, the concept of the exact structural designation through imaging is challenging from both a clinical and anatomic standpoint. On lateral radiographs or CT scans of the spine, the intervertebral disc is usually identified as a uniform soft-tissue opacity outlined by the raised epiphyseal rings and the subchondral bone plate [17]. Because we used high-quality contact radiography, we were able to directly visualize the disc and surrounding structures in fine detail. The demarcation of the nucleus from the annulus was clear, and the concentric arrangement of the annular fibers was readily seen. The apposing vertebral end plates were also visualized as radiodense osseous bands or arcs of a smooth or irregular configuration (Figure 7).

**Figure 8:**
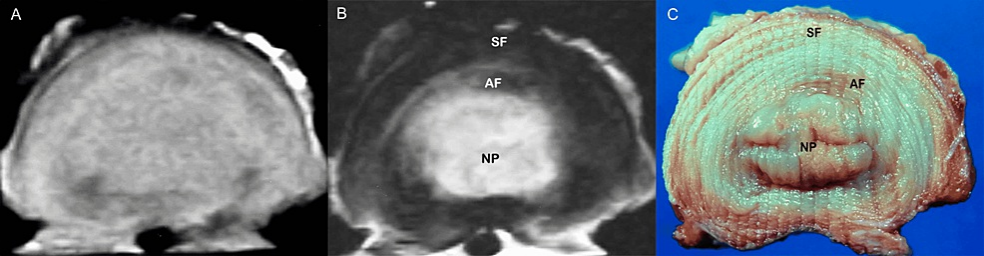
Normal disc, MR images and gross inspection. Axial T1-weighted (A) and T2-weighted (B) MR images show homogenous, intermediate signal intensity in the disc (A) and a central area of high signal intensity, the nucleus pulposus, surrounded by a peripheral portion of low signal intensity, the annulus fibrosus (B). (C) Axial anatomic section demonstrates the internal structure of the normal disc. Centrally located area of high signal intensity corresponds to the nucleus pulposus and the inner portion of the annulus fibrosus. NP: nucleus pulposus, AF: annulus fibrosus, SF: Sharpey fibers.

On the detailed evaluation of the morphologic anatomy characteristics of the intervertebral disc afforded by MR imaging, we noted that although in young individuals the nucleus pulposus is discernible from the annulus fibrosus, in middle-aged subjects and the elderly it is difficult to separate the nucleus from the annulus, and as such these components are considered to form a unicompartmental structure at large. On sagittal T1- and T2-weighted images, we have observed that the peripheral portion of the annulus fibrosus on the anterior surface of the intervertebral disc shows discrete hypointense signals, denoting stout fibrous bands in the anterior third of the disc. Sequential sagittal images may reveal the site of origin of Sharpey fibers at the most anterior portion of the discovertebral junction. Individual Sharpey fibers are not visible on MR images, however. We noted that axial images delineate the elliptical surface of the intervertebral disc to its full extent. Axial T1-weighted images demonstrate homogenous, intermediate signal intensity in the disc. On T2-weighted images, the disc exhibits a central area of high signal intensity surrounded by a periphery of low signal intensity. On the corresponding anatomic sections, this central region represents both the nucleus pulposus and the opal-whitish, glistening fibrocartilaginous inner zone of the annulus fibrosus. The peripheral zone of the annulus fibrosus is composed of collagenous fibers that blend imperceptibly with Sharpey fibers, which is also well demonstrated (Figure 8).

**Figure 9:**
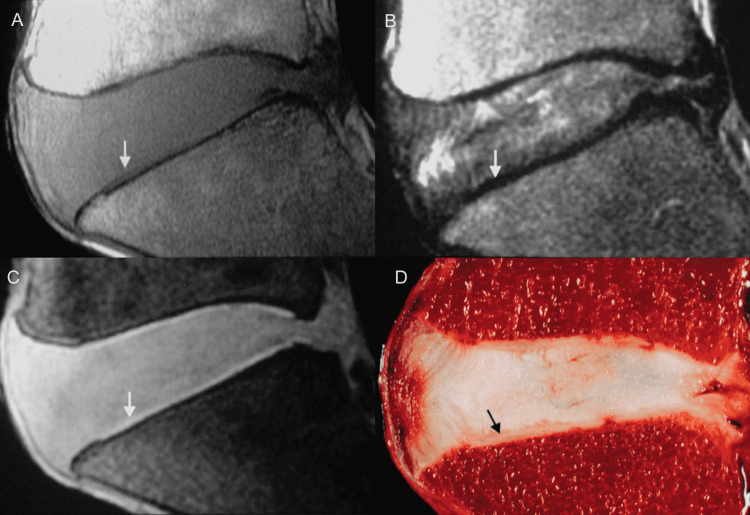
Cartilaginous end plates. (A) Sagittal T1-weighted MR image shows cartilaginous end plate (CP) (in portions) (arrow) with intermediate signal intensity. The deeper layer of CP investing subchondral bone is of low signal intensity owing to calcified cartilage, and differentiation between cartilage and bone is not possible. (B) Corresponding sagittal T2-weighted MR image shows cartilaginous end plate (CP) and subchondral bone of uniform, low signal intensity (arrow) compared to the intervertebral disc. (C) Fat-suppressed 3D-SPoiled Gradient-Recalled echo sequence (SPGR) MR image exhibits cartilaginous end plate (CP) as the distinct, continuous band-like structure of high signal intensity. (D) Corresponding sagittal anatomic section outlines the cartilaginous end plate (CP).

MR imaging in the sagittal plane readily outlines the vertebral end plates as relatively thick and uniformly hypointense structures situated between the vertebral marrow and the disc. The vertebral end plates appear slightly bowed, and the cartilaginous end plates conform accordingly to the depression of the bony end plates. On T1- and intermediate-weighted images, the superficial layer of the cartilaginous end plates exhibits intermediate signal intensity, although their deep layer attached to the subchondral bone shows a low signal intensity and is not generally discernible from the underlying bone. On T2-weighted images, both structures show low signal intensity, and differentiation between cartilage and subchondral bone is not feasible. Surface coils are basic to achieving high-resolution images with thin sections (2 mm) and a small field of view (8-10 cm). Because of the fine structure of the cartilaginous end plates, improved resolution and detailed sharp definition of the contours of the vertebral end plates were achieved with the use of a surface coil and gradient-echo sequences, in reference to previous postmortem studies [24] (Figure 9).

**Figure 10:**
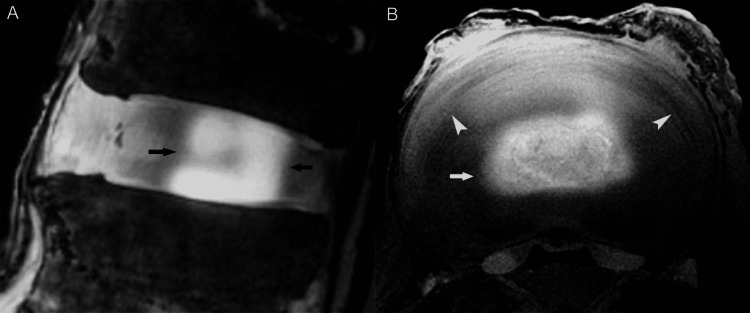
Normal disc, MR discography. (A) Sagittal MR discography image reveals the bilobular configuration of the normal nucleus pulposus (arrows). (B) Axial MR discography image shows a collection of gadolinium-containing contrast material in the center of the disc, within the nucleus (arrow). Note the concentric, onion-like arrangement of fibers in the annulus fibrosus.

The value of MR discography (a minimally invasive procedure involving intradiscal injection of gadolinium-containing contrast material) in evaluating disc morphology and abnormalities directly related to discogenic pain has been studied [25]. Complementary MR discography (n=28) readily identified the nucleus pulposus as a distinct structure located somewhat posterior to the midline of the disc. On the sagittal discographic images, the physiologic nucleus is visualized as two parallel plate-like collections of contrast material communicating posteriorly, whereas on the axial images the intact, normal nucleus appears as a lobular structure filled entirely with the intradiscal injected contrast material [25] (Figure 10).

We realized that no other imaging method affords direct visualization of the nucleus pulposus itself, a finding that may indeed prove of diagnostic value in the investigation of discogenic pain in living subjects.

**Figure 11:**
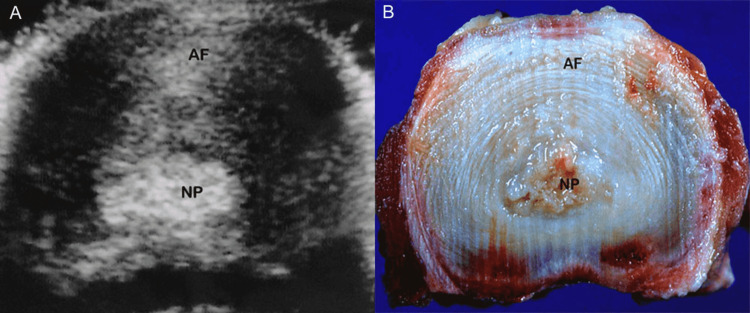
Normal disc, ultrasound. (A) Axial high-resolution sonographic image outlines the hyperechogenic nucleus pulposus (NP). There are numerous fine echoes in the annulus fibrosus (AF) at the periphery of the disc. (B) Corresponding anatomic section in axial plane delineates nucleus pulposus (NP) engulfed by annulus fibrosus (AF).

Sagittal high-resolution sonographic images (n=35) acquired with 10-MHz linear array transducers demonstrate the normal echogenic structure of the disc, sandwiched between the vertebral bodies. The nucleus pulposus appears relatively isoechoic or hyperechoic to the annulus fibrosus. On the axial plane, the echogenic nucleus pulposus is situated in the center of the disc, and a distinction between the nucleus and the annulus is feasible. Axial images show numerous, concentric fine echoes in the outer portion of the annulus fibrosus, corresponding to its multilayered architectural structure [26] (Figure 11).

## Discussion

Although post-mortem studies are fundamental to further understanding of the imaging findings as reflected by the anatomic sections, this study in cadavers has some weaknesses. First, we used standard MR imaging sequences to delineate basic anatomy in great detail, similar to that revealed in the anatomic studies. Dynamic contrast-enhanced imaging, spectroscopy, diffusion-weighted imaging, or other MR imaging techniques that are utilized in living subjects aimed at biochemical tissue analysis and functional assessment of anatomy in the spine are not actually applicable in our study material [5,6,25,27-30]. The imaging protocols conducted in autopsy specimens used technical parameters aimed at a limited portion of the body, and such protocols proved useful to direct assessment of these structures, however. Second, many specimens were taken from the cadavers of elderly humans; therefore, some of the investigated structures may have been subjected to degeneration already. At this time, few reports have discussed the anatomy of the intervertebral disc and the discovertebral segment. To our knowledge, the present study contains the largest number of cadavers studied with MR imaging-anatomic correlation. This study is unique not only in a large number of cadavers and discovertebral segments analyzed but also in the utilization of high-resolution radiography of sections of the specimen (slab radiographs) rather than of the entire specimen, a technique that would be expected to increase detailed visualization of sectional anatomy around the intervertebral disc. The study also engages imaging techniques dedicated to the disc, including MR discography and high-resolution disc sonography, to delineate anatomy. Finally, focused evaluation of the complex anatomic structure of the lumbar intervertebral disc and discovertebral segment in cadaveric specimens is fundamental to understanding the mechanism of disease processes in patients presenting with low back pain.

## Conclusions

The anatomy of the intervertebral disc and the discovertebral segment is quite complex. The results of our imaging-anatomic-pathologic correlative study provide insights into the internal structure of the intervertebral disc. We have concluded that good-quality radiographs and CT images are useful in the depiction of the bony end plates. Distinct components comprising the intervertebral disc can be depicted with MR imaging in detail similar to that revealed in anatomic studies of cadavers. MR discography is the only imaging technique that allows a dedicated depiction of the nucleus pulposus. An understanding of the anatomy of the intervertebral disc may provide a basis for further characterization of alterations in discal structure, igniting symptoms and disability.
